# Practitioner-Centered Design: A Case Study of a Human–Computer Interaction–Clinician Collaboration on the Development of Prototype Virtual Reality Environments for the Treatment of Posttraumatic Stress Disorder and Moral Injury in Health Care Workers

**DOI:** 10.1177/29941520261419919

**Published:** 2026-04-23

**Authors:** Jonathan I. Segal, Michael L. Turman, Mariel Emrich, S. Isabelle McLeod Daphnis, Samuel Rodriguez, JoAnn Difede, Andrea Stevenson Won

**Affiliations:** 1 Cornell University, Ithaca, New York, USA.; 2 Weill Cornell Medicine, New York, New York, USA.; 3 University of Connecticut, Storrs, Connecticut, USA.

**Keywords:** extended reality for trauma and stressor-related disorders, extended reality in nursing, human-centered design in medical extended reality, stakeholder adoption

## Abstract

The development of virtual reality–based psychotherapies requires interdisciplinary teams of subject matter experts, including technical and clinical experts. Such teams benefit from a “bridge”—either an existing team member with knowledge of multiple disciplines or, in the absence of a bridge member, a focused understanding of the norms and content of other involved fields. This article explores a collaboration between technical and clinical professionals developing immersive environments for treating posttraumatic stress disorder and moral injury in health care workers. This type of collaboration can generate valuable insights that guide the development of effective and targeted treatments. This article details the processes of establishing communication, identifying and overcoming differences in work expectations and knowledge, and how the team addressed critical issues during this process.

## Introduction

Human–computer interaction (HCI) is an interdisciplinary field by nature. For example, developing virtual reality (VR) applications is an interdisciplinary process, as these applications often aim to digitally represent objects and events drawn from the physical world, requiring domain experts to collaborate with experts in VR to ensure accuracy. VR in medicine has expanded rapidly in recent years, propelled by the emergence of affordable, user-friendly VR technologies and a growing body of clinical studies demonstrating the efficacy of VR-based interventions across diverse clinical settings.^1–3^ This article details a collaboration between technical and clinical professionals to develop VR environments to treat posttraumatic stress disorder (PTSD) and moral injury in health care workers (HCWs). Our contribution consists of formative collaboration and design practices, but does not report patient outcomes, feasibility metrics, engagement, dropout, or long-term effects in this phase.

Although such cross-disciplinary collaborations have benefits, they also raise challenges due to differences in areas of expertise, language/semantics, and communication norms between the two groups. These concerns are best addressed through a bridge, a person who can translate ideas between fields and help the team align expectations, terminology, and goals. A bridge may be a single member with experience in both areas, or a role collectively filled when all members make the effort to understand each other’s work practices. Throughout this article, we use “bridge” to mean a person who connects groups by translating terms, coordinating work, and maintaining a shared understanding.

Here, we use an HCI–clinician collaboration on developing a VR environment for PTSD and moral injury in HCWs as a case study. Developing VR therapies requires both clinical knowledge of PTSD and moral injury treatment and technical skill in creating immersive virtual environments. This collaboration benefited from the experience of two members who served as bridges, acting as translators who connected clinical goals with technical design decisions. The principal investigator (PI) on the clinical side has had extensive experience developing prototypes of VR simulations, developing and implementing the treatment applications of VR, working in interdisciplinary collaborations in academia and industry, and direct clinical experience providing patient care. The PI on the HCI side had experience in clinical settings, in addition to previous collaborations with clinicians. In this article, we characterize three stages of the collaboration and describe four types of challenges the team faced and solutions they identified.

**FIG. 1.**
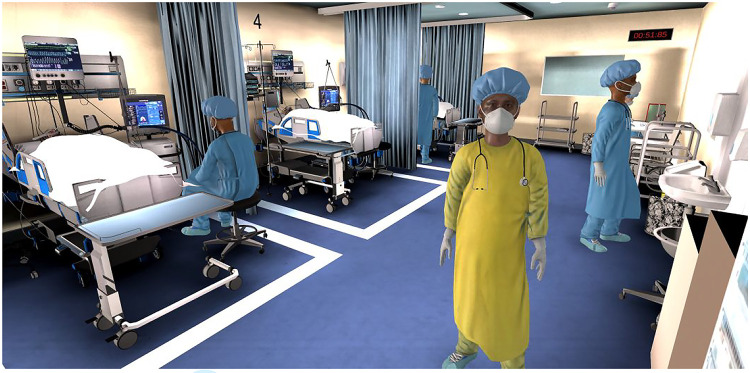
Image of the virtual ICU environment created using an existing ICU model for the project.^4^ The environment has hospital beds, health care workers in full personal protective equipment, and patients who are intubated.

Our contributions are as follows:

1. We describe a case study of developing a VR module for pilot testing in a patient population of HCWs. This environment is shown in Figure 1.
2. We make recommendations for collaborations between health care experts and HCI researchers and generalize these to collaborations on building VR environments more generally.

## Related Work

Below, we summarize work on HCI–clinician teams, collaborative design for VR, and VR for PTSD, focusing on findings that directly inform our case.

### HCI–clinician research collaborations

Projects that combine clinical and technical expertise report recurring challenges around misaligned goals, timelines, and evaluation standards.^5–7^ Collaboration quality is strongly tied to boundary-spanning communication, shared terminology, and clarity about clinical constraints such as privacy and workflow.^8,9^ Practical barriers in hospital settings, including Institutional Review Board (IRB) processes and security requirements, often elongate iteration cycles and limit access to end users.^10,11^ Our account focuses on how a designated bridge role and lightweight feedback materials (e.g., screenshots, short videos, and change logs used during iteration) helped reduce these frictions in practice.

### Conceptual framework for collaboration

**FIG. 3.**
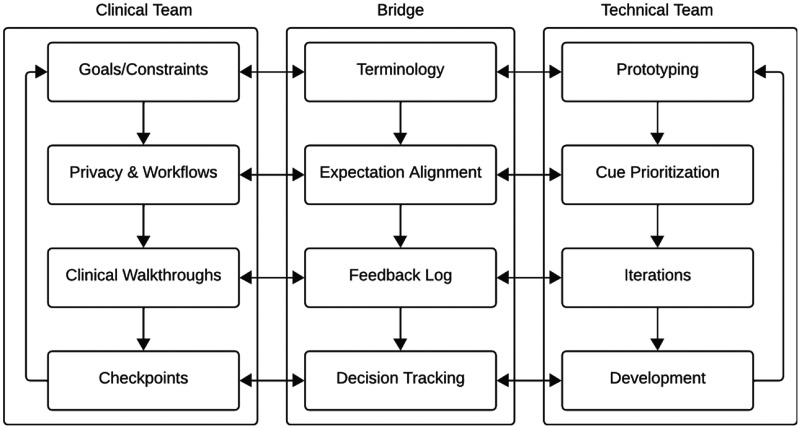
Interdisciplinary collaboration workflow with three lanes: clinical team, bridge, and HCI team. Boxes show key activities per lane with vertical flow; horizontal arrows show handoffs and feedback. HCI, human–computer interaction.

Our analysis draws on three perspectives from the literature on interdisciplinary teamwork. First, we adopt the idea of boundary-spanning roles from team science, which emphasizes explicit coordination and translation across domains with differing vocabularies and norms.^6,8^ Second, frameworks that highlight the importance of role clarity, feedback cycles, and compatibility between technical systems and clinical workflows.^9^ Finally, we incorporate communication alignment principles from human factors and organizational psychology, which stress shared mental models and iterative feedback as predictors of effective collaboration. We apply these lenses descriptively rather than theoretically, using them to interpret how bridge members facilitated shared understanding, structured iteration, and reduced friction between technical and clinical subteams. Figure 3 maps the collaboration structure of roles and handoffs. We emphasize three constructs throughout: role clarity and boundary spanning (team science), compatibility with clinical workflow (implementation science), and shared mental models (human factors).

### Collaboratively designing VR experiences

Interdisciplinary teams frequently adopt collaborative or co-design methods to bring clinical knowledge into early prototyping.^12,13^ Techniques include structured co-design sessions, quick iterative builds, and visual prompts that make assumptions explicit.^14,15^ Prior VR efforts show these methods can accelerate alignment on what details matter for fidelity without overinvesting in early asset polish.^16,17^ We follow this pattern and emphasize neutral placeholders and cue prioritization to keep stakeholders focused on behavior and flow.

### Application of VR for PTSD and moral injury treatment

Exposure therapy (ET) is an efficacious psychotherapy treatment for PTSD.^1^ ET involves repeated reengagement with the trauma memory to facilitate emotional processing and reduce cognitive and behavioral avoidance.^18^ Despite its efficacy, some individuals may experience difficulty engaging and immersing themselves in imaginal exposure, limiting the therapeutic gains.^19^ VR has emerged as a tool to facilitate engagement during exposure, and Virtual reality exposure therapy (VRET) has demonstrated efficacy as a treatment for PTSD.^3^ VRET immerses patients in controlled, trauma-relevant simulations to activate the trauma memory, support therapeutic processing, and reduce avoidance.^3,20^ The multisensory nature of VR may help patients access trauma-relevant memories and emotions more readily than traditional imaginal exposure.^20^

Early work using VR to treat PTSD in individuals affected by September 11, 2001, was pioneered by Difede and colleagues.^2,20,21^ This study set the framework for two decades of research developing and testing VR prototypes in the treatment of PTSD for various types of trauma, including the World Trade Center disaster^2,20,21^ and combat exposure,^22^ generating robust data from numerous randomized controlled trials that established VRET as an efficacious treatment for PTSD. Further, a recent study suggests that VRET may be more efficacious for PTSD patients with comorbid depression.^22^ This finding is particularly important given that comorbid depression is a significant predictor of worse outcomes with PTSD and co-occurs in about half of individuals who carry a primary diagnosis of PTSD.^23^

Although VRET has demonstrated efficacy for the treatment of PTSD in a range of diverse populations, its use remains understudied in HCWs. HCWs are routinely exposed to human suffering and death throughout their occupational duties and thus experience elevated rates of psychiatric conditions, such as PTSD.^24–27^ Many HCWs also struggle with moral injury, a form of psychological distress that can result from situations when they witness, participate in, or are unable to prevent actions that violate their values that are often due to institutional- or resource-based constraints. Although the use of VR to address moral injury is less studied than its application for PTSD, early VR simulations for moral distress in HCWs suggest that immersive scenarios can help clinicians reflect on ethically challenging events and coping strategies.^28,29^ Moral injury typically arises when individuals perceive that they have violated deeply held ethical beliefs or were constrained from acting according to them.^30^ VR can support therapeutic goals for moral injury by enabling guided reexposure to morally salient scenarios, structured perspective taking across roles, and reflective narrative reconstruction with clinician support. In our intensive care unit (ICU) module, these affordances informed design choices such as controlled replay of critical decisions, role-based scene variation, and neutral cues to foster reflective distance and facilitate cognitive restructuring. We view these as design hypotheses to be explored in later pilot studies.

In this case study, we focus on design and collaboration choices that improve contextual fidelity for ICU scenarios and on documenting the cross-disciplinary workflow that prepares for a feasibility pilot. We do not report patient outcomes in this phase. These mechanisms directly informed our ICU module choices (e.g., controlled replay of ethically salient moments and role-based scene variation), which we detail in the Case Study section.

## Case Study—PTSD and Moral Injury Module

The aims of the VR module developed by our team were to: (1) develop personalized virtual environments targeted to HCWs suffering from PTSD and related comorbidities, such as moral injury resulting from the COVID-19 pandemic, and (2) prepare materials and procedures for a future pilot study evaluating feasibility and clinical outcomes.

One long-term aim of the project was to incorporate personalization of both the patient-avatar and other avatars in the scene. In this case, personalization refers to creating an avatar that resembles the person’s physical body.^31^ In the current design and prototyping phase, we implemented and tested this capability with research staff rather than patients.

Through personalization, we aimed to increase a patient’s sense of body ownership and make them feel more present. This would facilitate further development of interventions targeted to the common comorbidities of PTSD, such as moral injury and depression.^31^

Allowing patients to customize therapeutic content may support motivation and perceived treatment fit. Meta-analytic work suggests that preference-congruent care is associated with modest improvements in outcomes across psychotherapy contexts.^32^ Evidence on personalization reducing dropout or improving long-term outcomes in PTSD specifically remains limited; we therefore treat personalization as a design hypothesis to be evaluated in future clinical studies rather than a demonstrated effect in this study.

Adding avatar personalization (letting patients match the avatar to their body) was novel for VRET in our setting. It also surfaced differences in which details mattered most, showed hardware limits we had to work within, and raised privacy questions, which we discuss below.

Clinicians noted that team composition and role visibility are central to ICU realism and to clinical narratives related to PTSD and moral injury. To support contextual fidelity and role salience without increasing risk, we adjusted nonself avatars at the level of role, posture, and task sequencing (e.g., a nurse at bedside or a respiratory therapist at the ventilator) rather than identifiable facial geometry. The goal is to cue teamwork dynamics and environmental meaning making while keeping identity features neutral.

### Team structure

Author/team member experience varied from subject matter experts working in both technical and medical fields for decades to undergraduate research assistants. HCI collaborators included a PI with extensive experience in VR research and many projects with a health care focus. A PhD student led a team of undergraduate research assistants on the project with a range of VR experiences. Clinical collaborators consisted of a PI housed at a medical college with extensive experience developing virtual environments, implementing clinical research trials, and leading interdisciplinary teams and medical VR projects; a postdoctoral fellow in psychology who had previously worked as an emergency medical technician; and a clinical psychology PhD student specializing in trauma and health psychology research. One postbaccalaureate coordinator, jointly employed by both the clinical and HCI labs, had experience in clinical VR research and served as a third bridge, facilitating communication and shared understanding between teams. The clinical team was also uniquely positioned for this collaboration due to firsthand experience with the subject environment, as several team members had personal experience working in hospitals during the early days of the pandemic.

The HCI and clinical groups had separate and joint team meetings. The channels between this team and the clinical team remained narrow, and many of the undergraduate research assistants (RAs) were relatively unaware of the clinical side of the project, occasionally leading to development decisions that required later clinical correction. However, this improved efficiency by minimizing meetings. When undergraduate RAs expressed interest in the clinical side of the project, they then needed to be onboarded and receive training on team norms, which helped develop them into bridge roles and prevented future misalignment.

Challenges arose during the project due to team dynamics and logistical issues. Both PIs belonged to teaching institutions, and members changed as students either graduated or moved on to other projects. This turnover caused the loss of project knowledge and required time to train new members. This took time and resources as new members had to be brought up to speed on the project’s goals, methodologies, and status.

Several forms of communication were critical to the project. The team used both weekly meetings and an ongoing email thread for sharing general updates. The HCI and clinical PIs communicated via regular remote meetings and texts. The HCI researchers also communicated via Slack. At weekly meetings, the HCI team demonstrated the current state of the application. The clinical team gave feedback on the prototype and suggested ways to make it easier to apply and use in patient care settings. Build updates and video demonstrations of the environment were regularly shared with the team via email. A detailed, shared log contained all changes to the environment as the build progressed. The application and versions were saved on GitHub, and changes were tracked using the GitHub release system, but all changes were also listed when sharing the new application over email to the clinical team, since they were unfamiliar with GitHub. In-person meetings were rare due to distance. While digital communication tools like Zoom worked well for screen sharing, face-to-face interactions were more advantageous when setting up equipment.

### Timeline

**FIG. 2.**
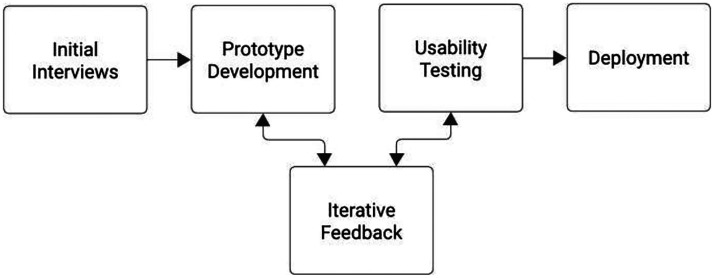
A diagram illustrating the timeline of the project.

The project began with a series of discussions and iterative feedback from medical experts across multiple disciplines (e.g., physicians, nurses known to the medical college–based team) to identify key visual and aural elements (Fig. 2).

Interviews were conducted to receive feedback on the design of the VR application. Stock images were presented to frontline HCWs, and a process of iterative feedback was used to elicit feedback on what important elements were necessary to evoke a feeling of being in a COVID-19 ward during the height of the lockdown. These interviews were conducted with those within the medical college PI’s personal network. The PI and several team members at the medical college led the interviews, drawing on extensive prior experience building VR prototypes, particularly due to the potential distress these conversations could elicit.

The HCI team then built the prototype environments, beginning with preexisting assets and incorporating the customization features. Next, the HCI team worked with the clinical team to develop desktop versions of the environments, incorporating iterative feedback on the visual and audio aspects of the virtual environment, and the virtual clinicians shown in the scene. The team then deployed the environment to different headsets to test usability.

### Formative clinician feedback and design impact

The primary evaluation data came from the clinical members of our team, who provided detailed feedback during iterative walkthroughs of each build. Their input focused on three main areas: (1) ensuring realism in clinical cues such as alarm cadence, monitor readouts, and staff behavior; (2) using neutral placeholders to reduce distraction during early scene development; and (3) calibrating avatar personalization options to respect patient comfort. This feedback led us to swap detailed placeholders for mannequins, fine-tune alarm timing, adjust room props for clinical usability, store all sensitive data locally, and create a short onboarding guide for consistent deployment.

### Mismatches in clinical/HCI expectations

#### Differences between clinical and HCI settings

Differences in protocol between clinical and HCI settings became apparent at the beginning of the project. A major area where protocol differences appeared was in expectations for privacy, formality, and communication. Clinical team members prioritized Health Insurance Portability and Accountability Act (HIPAA) compliance and formal professional norms, while HCI members tended to use more informal communication styles. These differences were quickly identified and incorporated into onboarding materials so that new team members could adjust to the interdisciplinary setting without confusion.

#### Integrating content and development expertise

Though adept at creating immersive VR experiences, the HCI researchers lacked knowledge about the nuances of an ICU setting needed to build a virtual environment that would serve as a platform for the effective treatment of PTSD and moral injury. Their primary focus was to ensure that the development was feasible and that the environment was stable. Given the clinical team’s extensive experience developing and testing VR environments in multiple medical settings, they were uniquely suited to bring insights about the real-world appearance and dynamics of an ICU that needed to be included in a virtual environment to provide clinically effective results for this patient population. Thus, the clinical team monitored and corrected environmental (i.e., medical equipment positioned unrealistically) and situational (i.e., unrealistic vital signs cycling) inaccuracies that would potentially distract patients or prevent a patient from engaging with the trauma memory. The confluence of these two sets of expertise was crucial in ensuring that the VR environment captured the essence of an ICU and remained technologically stable. However, sequencing this valuable feedback appropriately within the development workflow created several challenges.

For example, different perspectives arose on the use of placeholder elements. In development, using a placeholder element is often appropriate to block out scenes before focusing on more detailed aspects. However, inaccuracies can be highly salient to experts. For example, when composing the scene, a single generic doctor avatar was used multiple times as a placeholder to determine where “doctors” (agent-avatars) would be positioned and how these would be animated. Because diversity in assets is often a challenge in this space,^33^ the HCI team wanted to determine placement first before creating or modifying assets to represent a diverse workforce. However, the clinicians were correct in pointing out that the placeholder was unrepresentative of the population and would thus be distracting. This led to the insight that placeholder elements themselves needed to be selected to be as neutral or low-resolution as possible—in other words, rather than using a detailed model of a doctor as a placeholder, it would be better to use a featureless mannequin to block out position and animation, to clearly communicate that the details of appearance were to follow. Another suggested technique was to visually indicate which aspects would be targets for future customization. For example, the texture of the avatars could be removed so that they appeared as gray silhouettes instead of detailed full-color avatars.

Technical challenges and development priorities also needed to be clearly conveyed between teams. Given that resources are inevitably limited, the HCI team needed to convey the relative difficulty of modifying elements of the environment. With this information, the clinical team could then select the elements that were essential to representing a critical care environment. They could then discuss alternatives for time-consuming or computationally expensive features; for example, creating an image that could be used to simulate the contents of a shelf rather than individually modeling each object.

When prototyping, using existing assets as a basis for ideation was more efficient than asking HCWs to generate content from scratch. To facilitate this, the HCI team provided preliminary screenshots of environmental elements, which were then used as visual cues during Zoom discussions, and mutual understanding was refined using “on-the-fly” searches. The 3D environment for these prototypes was sourced from TurboSquid, an online model store, but HCI participants needed to guide clinical team members to focus on details that were harder to model, like the specific equipment, rather than quick, easy changes like the color of the walls or the configuration of the curtains. The clinical team had the knowledge of the essential sights, sounds, and behaviors needed to create appropriate animations, but needed to learn to convey highly specific details (and in some cases, source their own reference materials, such as ventilator sounds) rather than rely on the HCI team members’ lay understanding of the hospital environment. For example, when tasked with finding models of sick patients, the HCI team initially selected animations of patients coughing violently, not knowing that very ill COVID patients would be lying still due to sedation. In addition to visual attributes, care was taken to ensure that the sounds mimicked those of a real ICU, producing realistic alarms in an appropriate pattern, and footsteps and distant conversations outside the door to induce the feeling of presence in a larger hospital.

#### Technical limitations

The clinical team expressed the benefit of portable VR headsets. However, a more powerful nonportable system allowed for higher fidelity visuals to show clinicians how the environment would look and give the developer feedback on what to modify. Once the team decided to use a tethered headset to run the environment, it had to be properly stationed in the area where therapy would be conducted, which required materials to be developed to coach clinical teams in downloading and installing the new versions onto the headsets. The virtual environment had to be tested to ensure the VR headset was capable of running the application.

The environment was later optimized to allow us to deploy the system on a portable headset. Utilizing a portable headset allows for greater use of the system, but requires significant focus on the system’s performance, potentially taking much time. HCI team members also needed to balance accuracy and costs, including optimizing models. Thus, prioritizing which models needed to be highly detailed, and which could be rendered in less detail, for example, the contents of cabinets were a constant point of discussion.

#### Data and privacy

During development, we used scanned avatars of research staff to test personalization workflows; however, using patients’ actual faces would be protected health information. Thus, any avatar personalization technique, as with any software platform involved in this project, needed to be HIPAA compliant. In this phase, no patient-avatar scans were collected. The most tenable solution was to run the app on a local server, meaning it would never be uploaded to the cloud and would only be stored on lab devices. However, many consumer applications lack the capability or willingness to work with labs to ensure secure information storage.

Similarly, a rich information source about user experience in VR comes from the tracked movements of the participants, which creates a detailed record of what each user did and experienced in the virtual environment. A data recording system was developed to continuously record while the experiment runs, collect all the data from the headset’s position to the room’s state, and save it as a comma-separated values (CSV) file. However, this collection needs to comply with clinical regulatory concerns, notably HIPAA regulations, the local medical college’s IRB, and cybersecurity standards. Privacy and compliance issues were important to discuss at the beginning of the implementation of these applications to ensure that proper considerations could be accommodated within the given time frame.

To preserve privacy, all nonparticipant characters were represented with generic avatars whose appearance could not identify any real person. Optional self-avatar scans, when used, were stored only on encrypted local devices under IRB-approved access controls; no cloud storage was used.

## Discussion, Limitations, and Next Steps

Our collaboration developing VR therapies for PTSD and moral injury in HCWs highlighted key challenges in combining HCI and clinical expertise. Similar to prior work balancing technical and clinical goals,^8^ we focused on creating realistic scenes and enabling avatar personalization.^12,34^

Our case study on HCI–clinician collaboration emphasizes effective communication between diverse teams as essential for project success.^8^ The use of collaborative design methods allowed for active clinical team participation, ensuring our VR environments were both stable and accurate for clinical use.^34^ The VR environment development involved three key stages: (1) information gathering, which involved interviews with HCWs and required navigating cultural differences between clinical and HCI settings; (2) building, focused on balancing detail and efficiency in VR prototyping while ensuring technical accuracy and maintaining project momentum; and (3) deployment and testing, in which HCI and clinical experts worked together to make sure the headsets could run the app and that we met privacy and security standards for use in the hospital. Each stage highlighted the need for aligning technical capabilities with medical requirements, showcasing the benefits and challenges of working across teams in health care technology.

We interpret the bridge role and our cross-team practices through established collaboration and implementation constructs, including boundary-spanning roles in team science, communication, and workflow alignment from implementation frameworks (e.g., role clarity, feedback cycles, and compatibility). We use these constructs descriptively to explain why early bridge designation, shared terminology, and cue prioritization improved fit between technical feasibility and clinical routines in our case.

Insights from clinician walkthroughs highlighted the importance of selectively adding detail to virtual environments. Clinicians emphasized that realism cues tied to ICU practice (e.g., alarm variability, monitor logic) were most important for perceived fidelity. These inputs informed our cue prioritization for subsequent versions of the environment; no patient outcomes were collected in this phase.

Successful collaboration required learning about each field through short immersive exchanges, such as HCI team visits to the hospital setting. Clear onboarding and communication helped members understand each other’s roles and priorities. Establishing group norms that value all members’ expertise fostered mutual understanding and belonging across teams.

We highlight the importance of designating a bridge to coordinate communication, clarify expectations, and maintain shared understanding across technical and clinical teams. Early alignment on communication patterns, such as how often teams meet and how feedback is exchanged, shared terminology, and clinician involvement in early prototypes, helps prevent missteps and keep projects cohesive. These practices set the foundation for the more detailed, transferable steps outlined below.

What makes this research unique is that it reflects the essence of HCI, as it is adaptive and aims to serve an ever-changing environment. With the effects of COVID-19 on HCWs and the broader community, it is vital for the medical community to work to ameliorate the hazardous effects of the pandemic. A significant aspect of this evolution involves caring for the workers who provide care. With systems such as the ones we are working on, it is not only HCWs that benefit from the technology but also all patients that come into contact with these workers.

### Guidelines and roadmap for XR–health collaborations

We summarize the collaboration workflow in Figure 3, then distill practical steps for future teams:

- Establish a clear bridge role early in the project and define its responsibilities so translation, decision tracking, and cross-team alignment happen continuously rather than on an *ad hoc* basis.
- Align expectations using shared artifacts such as simple visuals, a short terminology glossary, and a living change log that links feedback to resulting actions to reduce semantic drift.
- Begin with low-fidelity builds that use neutral placeholders to keep attention on workflow, timing, and safety rather than surface detail.
- Prioritize realism cues that clinicians identify as most consequential for perceived fidelity, such as monitor logic or alarm cadence, and implement those elements first.
- Iterate in a visible and accountable manner so every comment is either addressed or documented as a tradeoff, which sustains trust and forward momentum.
- Address privacy and regulatory constraints at the start of the collaboration, and keep data pathways minimal and local when possible to simplify compliance.
- For moral injury scenarios, design scenes to support perspective taking and reflective meaning making rather than straightforward exposure, using role variation and controlled replay to scaffold discussion.
- Use structured communication, ethical awareness, and staged fidelity as the foundation for later feasibility and outcome studies.

## Conclusion

Across extended reality for health (XR–health) projects and, more broadly, any collaboration between HCI teams and domain experts, a dedicated bridge role can improve clarity and alignment. Bridge members help translate terminology, surface constraints early, and maintain shared expectations as prototypes evolve. Our case shows that combining this role with structured communication reduces friction and supports more effective co-development. These practices can strengthen interdisciplinary teamwork in future XR–health initiatives and other technical–domain partnerships. A long-term goal of this ongoing project is to incorporate avatar personalization, but due to data safety concerns, discussions are still ongoing regarding how to ensure HIPAA compliance.

## Authors’ Contributions

J. I. Segal: Conceptualization; Methodology; Software; Investigation; Data curation; Analysis; Writing—original draft; Writing—review and editing; Project administration. Michael L. Turman: Conceptualization; Methodology; Investigation; Resources; Writing—review and editing. Mariel Emrich: Investigation; Data curation; Analysis; Writing—review and editing. S. Isabelle McLeod Daphnis: Investigation; Data curation; Writing—review and editing. Samuel Rodriguez: Investigation; Data curation; Software; Writing—review and editing. JoAnn Difede: Conceptualization; Methodology; Supervision; Project administration; Writing—review and editing. Andrea Stevenson Won: Conceptualization; Methodology; Supervision; Project administration; Writing—review and editing.

## Notes

**Cite this article as:** Segal JI, Turman ML, Emrich M, McLeod Daphnis S I, Rodriguez S, Difede JA, Won AS (2026) Practitioner-centered design: A case study of a human–computer interaction–clinician collaboration on the development of prototype virtual reality environments for the treatment of posttraumatic stress disorder and moral injury in health care workers, *Journal of Medical Extended Reality*, 2026, 3, 29941520261419919, DOI: 10.1177/29941520261419919.
